# Advanced triage protocols in the emergency department: A systematic review and meta-analysis^*^

**DOI:** 10.1590/1518-8345.5479.3511

**Published:** 2022-03-11

**Authors:** Cecilia Biasibetti Soster, Fernando Anschau, Nicole Hertzog Rodrigues, Luana Gabriela Alves da Silva, André Klafke

**Affiliations:** 1 Grupo Hospitalar Conceição, Gerência de Ensino e Pesquisa, Porto Alegre, RS, Brasil.; 2 Grupo Hospitalar Conceição, Porto Alegre, RS, Brasil.; 3 Pontifícia Universidade Católica do Rio Grande do Sul, Escola de Medicina, Porto Alegre, RS, Brasil.; 4 Universidade Federal de Ciências da Saúde de Porto Alegre, Porto Alegre, RS, Brasil.; 5 Santa Casa de Misericórdia de Porto Alegre, Hospital Dom João Becker, Gravataí, RS, Brasil.

**Keywords:** Triage, Biomedical Technology Assessment, Emergency Medical Services, Advanced Practice Nursing, Evidence-Based Nursing, Systematic Review, Triagem, Avaliação da Tecnologia Biomédica, Serviços Médicos de Emergência, Prática Avançada de Enfermagem, Enfermagem Baseada em Evidências, Revisão Sistemática, Triaje, Evaluación de la Tecnología Biomédica, Servicios Médicos de Urgencia, Enfermería de Práctica Avanzada, Enfermería Basada en la Evidencia, Revisión Sistemática

## Abstract

**Objective:**

To evaluate the effectiveness of using advanced triage protocols on the length of stay, safety and satisfaction of the patients and professionals in the emergency department.

**Method:**

A systematic review with meta-analysis of randomized clinical trials included in the Embase, BVS, PubMed, CINAHL, Cochrane Library databases and in the gray literature, using Review Manager 5.4. Studies that analyzed length of stay in their outcomes were included and the studies excluded were those that considered other triage protocols.

**Results:**

26,672 peer-reviewed studies were found and ten were included in the meta-analysis. For the patients’ length of stay, seven studies were included in the meta-analysis (n=8,229), showing a 36-minute reduction (-0.36[-0.55;-0.17] p=0.002), a result with low certainty of evidence, favorable to the intervention, varying between -0.53 (-0.81;- 0.25) and -0.29 (-0.50;-0.07) in the analysis of the subgroups. Regarding the exams requested, five studies were included (n=2,270), indicating that there is no significant difference between the groups (*odds ratio* 0.94 [0.64;1.38]). Four studies (n=6,094) analyzed the patients’ and the professionals’ satisfaction, indicating a favorable result to the intervention.

**Conclusion:**

The advanced triage protocol reduced length of stay in the emergency room without compromising care safety and quality, although more randomized clinical studies on the theme are needed. Registered in PROSPERO (CRD42019142640).

## Resumo

**Objetivo:**

Avaliar a efetividade do uso de protocolos de triagem avançada no tempo de permanência, segurança e satisfação dos pacientes e profissionais no serviço de emergência.

**Método:**

Revisão sistemática com metanálise de ensaios clínicos randomizados das bases de dados Embase, BVS, PubMed, CINAHL, *Cochrane Library* e literatura cinza, utilizando o *Review Manager* 5.4. Foram incluídos estudos que analisaram tempo de permanência em seus desfechos e excluídos estudos que consideraram outros protocolos de triagem.

**Resultados:**

Foram encontrados 26.672 estudos, revisados por pares, e dez foram incluídos na metanálise. Para tempo de permanência dos pacientes, sete estudos foram incluídos na metanálise (n=8.229), mostrando uma redução de 36 minutos (-0,36[-0,55; -0,17] p=0,002), resultado com baixa certeza de evidências, favorável à intervenção, variando entre -0,53(-0,81; - 0,25) e -0,29(-0,50; -0,07) na análise dos subgrupos. Quanto aos exames solicitados, cinco estudos foram incluídos (n=2270), indicando que não há diferença significativa entre os grupos (*odds ratio* 0,94 [0,64;1,38]). Quatro estudos (n=6094) analisaram a satisfação dos pacientes e profissionais, apontando um resultado favorável à intervenção.

**Conclusão:**

O protocolo de triagem avançada reduziu o tempo de permanência na emergência sem comprometer a segurança e a qualidade do atendimento, embora sejam necessários mais estudos clínicos randomizados sobre o tema. Registrado na PROSPERO (CRD42019142640).

## Resumen

**Objetivo:**

Evaluar la efectividad del uso de protocolos de triage avanzado en la duración de la estancia, seguridad y satisfacción de los pacientes y profesionales en el servicio de emergencia.

**Método:**

Revisión sistemática con metaanálisis de ensayos clínicos aleatorizados de las bases de datos Embase, BVS, PubMed, CINAHL, *Cochrane Library* y de la literatura gris, utilizando *Review Manager* 5.4. Se incluyeron los estudios que analizaron la duración de la estancia en sus resultados y se excluyeron los estudios que consideraron otros protocolos de triage.

**Resultados:**

Se encontraron 26.672 estudios, fueron revisados por pares y diez se incluyeron en el metaanálisis. Para la duración de la estancia de los pacientes, se incluyeron siete estudios en el metaanálisis (n=8.229), que muestran una reducción de 36 minutos (-0,36 [-0,55; -0,17] p=0,002), un resultado con baja certeza de la evidencia, favorable a la intervención, que osciló entre -0,53(-0,81;-0,25) y -0,29(-0,50;-0,07) en el análisis de los subgrupos. En cuanto a las pruebas solicitadas, se incluyeron cinco estudios (n=2270) que indican que no hay diferencia significativa entre los grupos (*odds ratio* 0,94 [0,64; 1,38]). Cuatro estudios (n=6094) analizaron la satisfacción de los pacientes y profesionales e indican que la intervención tuvo un resultado favorable.

**Conclusión:**

El protocolo de triage avanzado redujo la estancia en el servicio de emergencia sin comprometer la seguridad y la calidad de la atención, aunque es necesario que se realicen más ensayos clínicos aleatorizados sobre el tema. Registrado en PROSPERO (CRD42019142640).

**Highlights**

(1) Advanced Triage Protocol reduces Emergency Department overcrowding. (2) The Advanced Triage Protocol is a safe and effective practice in the Emergency Department. (3) Triage nurses are qualified to use Advanced Triage Protocol. (4) Increased satisfaction was identified among users submitted to the intervention. (5) Advanced Triage Protocol reduces patient length of stay in the Emergency Department.

## Introduction

Overcrowding of Emergency Services (ES) occupies the headlines of media vehicles daily and has shown exponential growth since the 1990s in several countries^1^ ^-^ ^2^. Since then, managers and specialists have been looking for alternatives that solve or attenuate this problem^3^ ^-^ ^6^.

Overcrowding of the ES is defined as an occupancy rate greater than 90% of their capacity, absence of beds, or patients allocated to inadequate spaces^3^ ^-^ ^4^. This situation reflects negatively on the quality, safety and efficiency of this service^5^. As it is a global health problem, overcrowding has been widely discussed, and alternatives have been developed and tested according to the local profile^3^ ^,^ ^5^ ^-^ ^8^. Although frequently related to the causes of ES overcrowding, with regard to the length of stay of the patient in the emergency services, there is no consensus that defines a safe permanence time, as well as there is no clarity of the criteria that define a prolonged length of stay. The parameters vary according to the locus and to the characteristics of the patients, which implies a broad variable of this measure, between 4 and 48 hours^9^.

In the search to solve overcrowding, triage with risk classification (RC), which derives from a military practice, began to be used in the ES. Despite a promising start of this strategy, in 1990 in Europe and in the mid-2000s in other countries, it did not address ES overcrowding, which is why Advanced Triage Protocols (ATPs)^1^ ^,^ ^5^ ^,^ ^10^ were proposed. 

The ATPs consist of standardized approaches, applicable to specific groups of patients, where the triage professional initiates diagnostic or therapeutic actions before the patients are seen by a physician. This movement allows reducing the length of stay of patients in the emergency service from the restructuring of the work process^11^. We highlight better results in the use of ATPs in emergency services that devote greater investment to the qualification and training of the triage professionals, placing this factor as a determinant of the safety and quality of this intervention^12^ ^-^ ^13^. 

According to a study conducted in Belgium, it is a resource that allows improving management of the patients and reducing their length of stay in the ES^1^. Some studies also suggest positive impacts on cost reduction^14^ and on care quality in the ES^13^. Therefore, the objective of this study is to evaluate the effectiveness of using ATPs on the patients’ length of stay, as well as on the safety and satisfaction of patients and ES professionals.

## Method

### Type of study

This is a systematic review (SR) of the literature with meta-analysis. It was registered in the International Prospective Register of Ongoing Systematic Reviews (PROSPERO) under number CRD42019142640 on 10/17/2019^15^, and was developed rigorously in the light of the guidelines recommended by the PRISMA statement^16^. 

### Search strategy

The development method consisted of the search for clinical randomized trials (CRTs) on the Embase, BVS, PubMed, CINAHL and Cochrane Library research platforms, in addition to the manual search in the reference lists of studies selected and in the gray literature to answer the following research question: Which is the effectiveness of using ATPs on the length of stay of patients in the ES? From the PICOS strategy (P: Patients who seek ES; I: Use of ATPs; C: Conventional triage; O: Length of stay of patients in the ES; and S: CRTs), the following search terms (MeSH) were used: *Randomized Controlled Trial*, *Triage*, *Hospital Emergency Services* and *Emergency*, as well as these free terms: *advanced triage, triage protocol* and *length of stay*, which, associated with Boolean operators (OR and AND), structured the search strategy.

### Search period

The study collection period was between December 2019 and March 2020.

### Selection criteria

The studies included were those of the CRT type that addressed the use of an ATP in the ES, and which measured in their outcomes the length of stay of patients in the ES. Studies relevant to the theme, found in the reference lists or gray literature, were also included. There was no language restriction in the research or regarding the publication period. Studies addressing non-face-to-face triage, ATPs initiated in the pre-hospital environment or that did not describe the patient’s length of stay among their outcomes were excluded. Studies where it was not possible to access complementary data after three attempts to contact the authors were also excluded. The articles considered irrelevant to this research were excluded in different selection phases, based on the criteria described.

### Study selection

The studies found were reviewed by four examiners, from the reading of titles and abstracts by peers, independently. Studies selected by only a pair of reviewers were included in the next phase. At this stage, the EndNote online reference manager was used. The lists of the studies selected in this phase were compared and agreement of the analysis was verified through the Kappa test. The agreement strength was defined as slight (from 0.00 to 0.20), fair (from 0.21 to 0.40), moderate (from 0.41 to 0.60), substantial (from 0.61 to 0.80) or almost perfect (from 0.81 to 1.00)^17^.

The second phase of the analysis consisted of the full reading of the articles by two researchers, independently. The aforementioned inclusion and exclusion criteria were considered, as well as the ethical aspects and the presence of clinically important results and objects for this review.

### Data treatment and analysis

Data extraction from the studies selected was conducted independently by two researchers. The following variables of each study were considered: first author, year of publication, place where the study was conducted, demographic data of the sample, type of intervention, type of comparison, patient’s length of stay and other outcomes. The results were summarized in a Microsoft Excel^®^ spreadsheet.

The meta-analysis was developed through the features of the Review Manager 5.4^18^ software. For the “length of stay” outcome, a continuous variable with random effect and calculation of the difference of the time mean values with a 95% confidence interval were considered, where Relative Risk (RR) below 1 means a favorable outcome to the intervention. For the “number of exams requested” outcome, the data were worked on under the proportion/risk difference, considered a dichotomous variable of random effect. Relative Risk (RR) was considered a measure of effect with 95%CI, where RR below 1 means that there is no difference between the groups. Conversions were performed to obtain means of the studies that presented their data in different format^19^. The outcome was considered statistically significant when p-value<0.05. If I^2^≤25, the studies were considered homogeneous, I^2^>25 and I^2^<75 classified the studies as having moderate heterogeneity and I^2^≥75, as with high heterogeneity. The subgroup analysis was developed when moderate or high heterogeneity was identified^20^. 

### Quality assessment

The analysis of the risk of bias was developed independently by two researchers based on the Cochrane Collaboration (RoB-2) tool. The following domains for internal validation were evaluated: selection bias, performance bias, detection bias; attrition bias and reporting bias, related to the following potential sources of bias: randomization, allocation concealment, blinding of participants and personnel, incomplete data of results, obfuscation of the evaluation of results and report selection of results^21^.

The GRADE system was used to evaluate the quality of the evidence and the strength of the health recommendations. This system provides explicit criteria to classify the quality of the evidence (classified into one of the following four levels: high, moderate, low and very low) that include study design, risk of bias, inaccuracy, inconsistency, indirect effect and magnitude of effect^22^. Two authors performed the analysis blindly and independently, through the GRADEpro platform^23^. 

## Results

**Figure 1:**
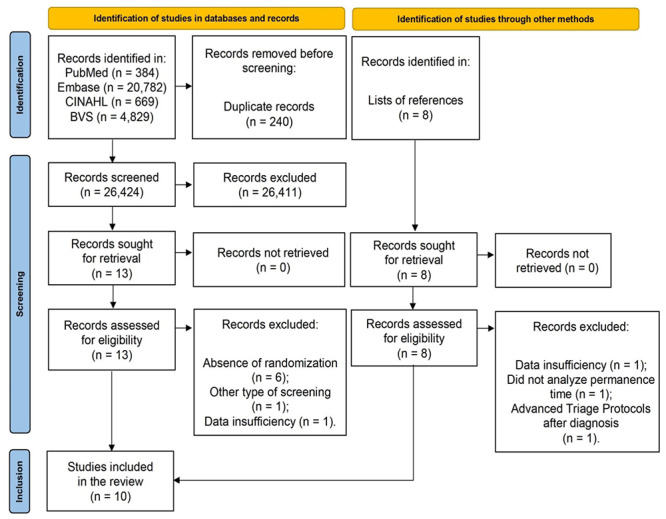
Flowchart corresponding to selection of the studies in the PRISMA 2020 format, showing the stages for the selection of CRTs of this systematic review. Porto Alegre, RS, Brazil, 2020.

According to what is presented in Figure 1, the literature review revealed 26,672 articles on ATPs in the emergency service (26,664 in the databases and 8 articles identified in the reference lists and in the gray literature). After the first analysis, 240 duplicate articles were excluded. Of the 26,432 related papers, 21 were listed as potentially relevant, keeping the focus on the object of this research. Of these, 11 were excluded in the second phase and 10 were selected for the qualitative analysis. The reasons for exclusion in this stage were as follows: using a different CRT design^24^ ^-^ ^29^, employing remote triage resources^30^, presenting insufficient data for analysis^1^ ^,^ ^31^ and consisting of ATPs for specific pathologies, applied at other times of hospitalization^32^. One study was excluded in this phase due to lack of data, without answer after three attempts to contact the authors^33^. Of the studies selected, nine were included in the meta-analysis. One study^34^ was excluded from the meta-analysis due to data inaccuracy, although its data were analyzed qualitatively. The articles selected for the analysis were published between 1996 and 2018. The Kappa index was К=0.73, identifying substantial agreement^34^.

**Figure 2:**
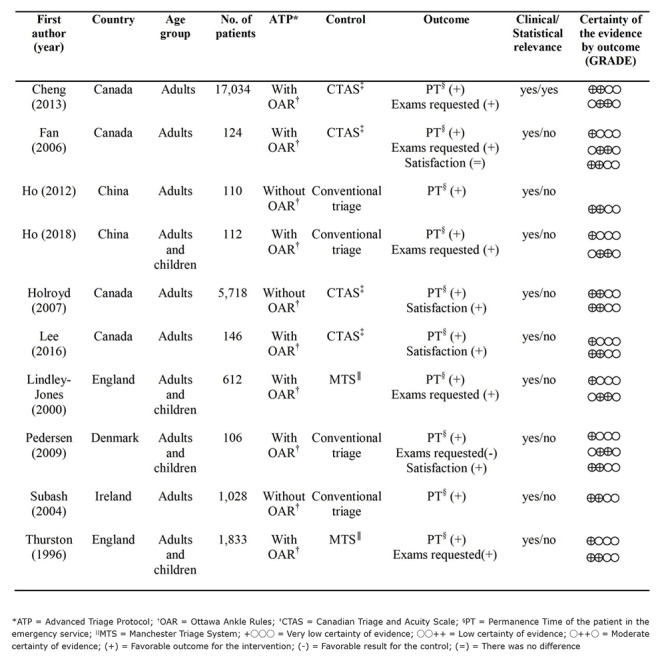
Characteristics of the studies included, considering study locus, participants, type of intervention, outcomes, study relevance and certainty of evidence. Porto Alegre, RS, Brazil, 2020.

Figure 2 shows the general characteristics of the studies listed. Four studies were conducted in Canada^33^ ^,^ ^35^ ^-^ ^37^. In addition, papers carried out in England^38^ ^-^ ^39^, China^40^ ^-^ ^41^, Ireland^42^ and Denmark^43^ were also included. Six studies^33^ ^,^ ^35^ ^-^ ^37^ ^,^ ^41^ ^-^ ^42^ restricted their population to adults; the others included adults and children in their samples^38^ ^-^ ^40^ ^,^ ^43^. Eight papers described their duration, which varied from eight days to one year ^(^ ^33^ ^,^ ^35^ ^-^ ^38^ ^,^ ^41^ ^-^ ^43^. Three studies included physicians and nurses in the triage of their interventions^35^ ^-^ ^36^ ^,^ ^42^. Four papers compared their interventions to the Canadian Triage and Acuity Scale (CTAS)^33^ ^,^ ^35^ ^-^ ^37^, another two made comparison with the Manchester Triage System^38^ ^-^ ^39^, and four studies did not describe the protocol used, but reported the use of some sorting order in the care provided. Six studies analyzed ATPs with imaging exams; of these, three^35^ ^,^ ^37^ ^,^ ^40^ used the Ottawa Ankle Rules (OAR)^44^. Another three studies evaluated more than one ATP, and one study assessed the use of bronchodilators in the triage^41^. Five papers described training of the triage nurses^37^ ^-^ ^38^ ^,^ ^40^ ^,^ ^43^.

The studies totaled 25,795 subjects, in 13 ES. One of them was carried out in the multicentric modality in four ES^39^. In addition to the length of stay of the patients in the ES, other outcomes were identified, such as: number of exams requested, number of hospitalizations, waiting time to be seen by the physician, number of patients who leave the ES without being examined, time for triage, time for medication, patients’ satisfaction and ambulance diversion time. All the studies herein listed recommended the use of ATPs in their conclusions, although only one of them found statistical relevance^35^.

**Figure 3:**
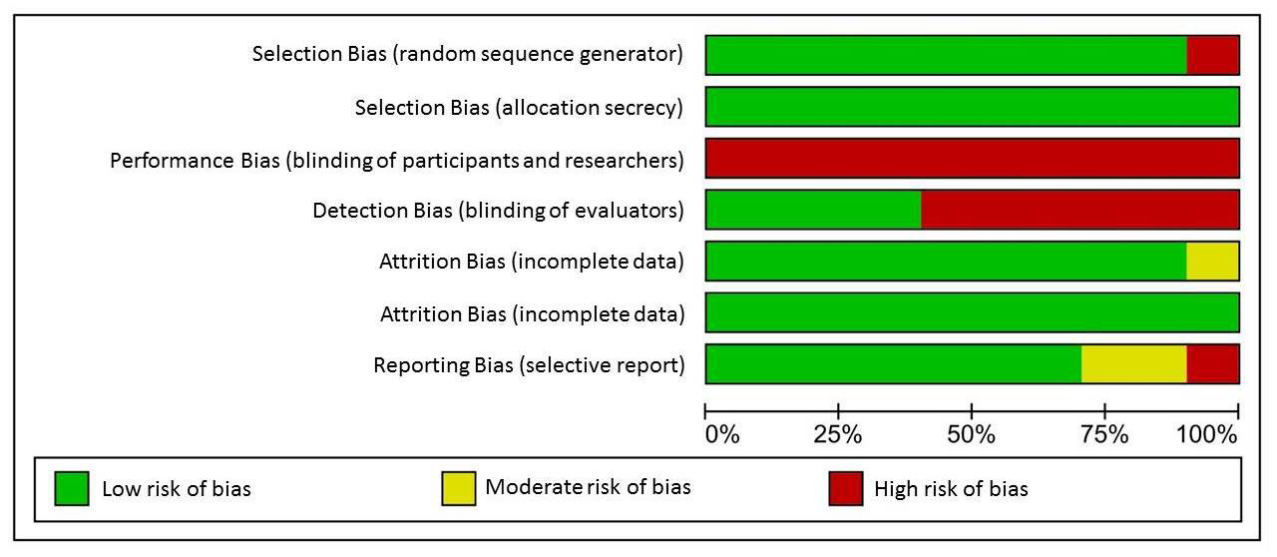
Summary of the risk of bias analysis, developed with the *Review Manager* 5.4 software. Porto Alegre, RS, Brazil, 2020.

Figure 3 presents the summary of the risk of bias analysis according to the Cochrane System (RoB2). Methodological quality was considered good among the studies included. Most of the studies have adequate randomization and allocation concealment. There was no reduction in quality, although no blinding of the participants was observed. In some studies, it was also identified that the evaluators were not blinded^33^ ^,^ ^35^ ^-^ ^37^ ^,^ ^39^ ^-^ ^40^ ^,^ ^42^ ^-^ ^43^. Other risks of bias were reported by the authors^38^ or are not clear. Due to the identification of only ten studies in this systematic review, despite the extensive search for studies, the risk of publication bias is considered, which was confirmed by the analysis of the funnel graph.

**Figure 4:**
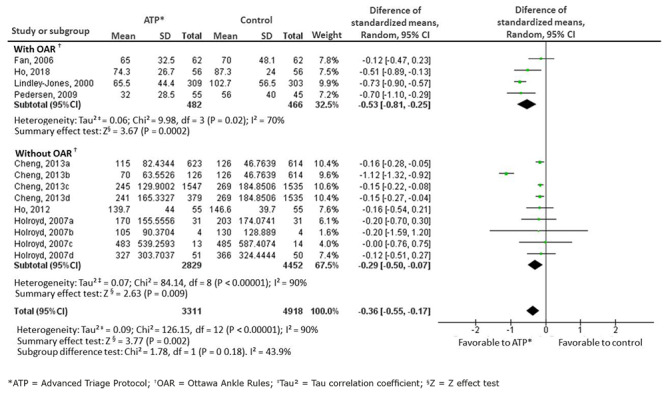
Forest graph showing the length of stay of the patients in the emergency service (in minutes). Porto Alegre, RS, Brazil, 2020.

Figure 4 presents the forest graph and meta-analysis of the main outcome of this systematic review, and the length of stay of the patients in the ES. A 36-minute reduction was identified (-0.36[-0.55;-0.17]), p=0.002, with the use of ATPs.

Given the high heterogeneity shown (I^2^=90%), subgroup analysis was performed, considering studies or strata of studies without imaging exams (without OAR): 9 studies/study strata, n=7,293, I^2^=90%; and with imaging exams (with OAR): 4 studies/study strata, n=948, I^2^=70%. Thus, the ATP shows a 29-minute reduction (-0.29[-0.50;-0.07]), with high heterogeneity (I^2^=90%), statistical relevance (p=0.009) and very low certainty of evidence, in the subgroup without OAR. In the subgroup with OAR, a 53-minute reduction (-0.53[-0.81;-0.25] p=0.002) is identified in the length of stay of the patients in the ES, with moderate heterogeneity (I^2^=70%) and low certainty of evidence. The means and standard deviation of length of stay were calculated from the conversion of the medians^19^, since most of the studies presented their data only in this format.

**Figure 5:**
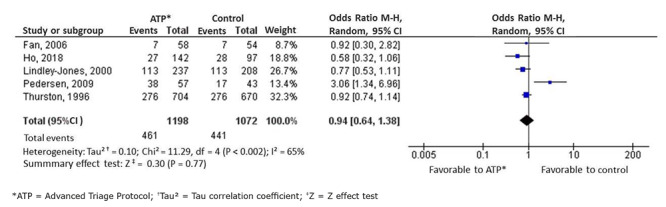
Forest graph showing the number of exams requested and fractures identified, comparing exams requested in the triage (ATP) and in the medical consultation (control). Porto Alegre, RS, Brazil, 2020.

Among the studies analyzed, five^35^ ^,^ ^38^ ^-^ ^39^ ^,^ ^41^ ^,^ ^43^ analyzed in their outcomes the number of exam requests with and without the use of ATPs. It was decided to include these data in the meta-analysis, presented in Figure 5. In a sample that comprised 2,270 requested exams, no difference between the groups is identified, obtaining a result of 0.94 OR (0.64; 1.38), p=0.7, with moderate heterogeneity (I^2^=65%) and moderate certainty of evidence.

Another outcome found in the analysis of the studies was the satisfaction of professionals and of patients subjected to the ATP in the ES, which was included in this study due to its relevance. As it was presented by the authors in different ways, it was decided to qualitatively analyze this outcome, based on the data presented in Figure 2.

A study included in this analysis^37^ evaluated the professionals’ satisfaction with the use of the ATP through focus groups held after the intervention and reported that 80% of the physicians and 90% of the nurses assessed the ATP as beneficial for the patients and that more than 70% of the professionals stated that the intervention promoted agility in the care provided. 

Other studies^33^ ^,^ ^43^ identified increased satisfaction. One of the studies^43^ performed this analysis separately based on questionnaires applied to the patients after the service, about their satisfaction and their desire to have access to the ATP in a new visit to the ES. The authors report that 89.2% of the patients who did not have access to the ATP by the ES would like to have been subjected to interventions initiated in the triage and 97.6% of the intervention group expressed the same desire. Another study^33^ evaluated this outcome through objective questionnaires, with answers on a numerical scale, applied by telephone to patients and professionals. With regard to the patients, satisfaction with the length of the appointment was better assessed in the group subjected to the ATP, with no difference between the groups in other aspects. Regarding the nurses’ satisfaction, the authors report that they feel comfortable using the ATP, believing that this intervention reduces the patients’ length of stay, although they expressed concern about the increase in workload in the triage.

Only one study^35^ found no difference between the groups in the patients’ satisfaction after the care provided. This outcome was analyzed using the Sun satisfaction scale, comprised of five levels, which analyzed users’ satisfaction based on their desire to return to the service and found no difference in the patients’ satisfaction after the appointment with ATP.

## Discussion

From the meta-analysis, this study found a 36-minute reduction (-0.36[-0.55;-0.07] p=0.002) in the length of stay of patients in the ES using the ATP. It was evidenced that there was no difference in the number of tests requested and in the patients’ and professionals’ satisfaction rates.

### Length of Stay

As for the patients’ length of stay, this study identified, with low certainty of evidence with high heterogeneity, findings that are similar to those found in other meta-analyses^12^ ^,^ ^45^. Heterogeneity is justified by two main factors: the difference between the types of ATP analyzed in each study and the difference between the samples. It was reduced by means of the analysis of the subgroups, categorized according to the type of ATP, in: with OAR (I^2^=70%) and without OAR (I^2^=90%). The possibility that substantial heterogeneity overestimates the study result is minimized by the fact that, it was in the more homogeneous subgroup (with OAR) that the highest reduction in length of stay was identified.

In the subgroup without OAR, three studies were included^36^ ^-^ ^37^ ^,^ ^41^, two of them subdivided into three strata, according to risk classification at the time of triage. Two studies^36^ ^-^ ^37^ analyzed an ATP that associated a physician’s face-to-face or telephone consultation to decide on the triage orders. One study^41^ analyzed an ATP consisting of initiation of the therapy with bronchodilators in the triage for patients with decompensated asthma. In this latter, the main finding reported was a clinical improvement in the patient when seen by the physician. When evaluating only ATPs that include therapeutic interventions, greater relevance is observed in care quality than in length of stay^12^.

In the subgroup with OAR, a 53-minute reduction in the length of stay of patients in the ES was identified (-0.53[-0.81;-0.25] p=0.0002). This intervention, as well as others, which also use diagnostic tests in the ATP, present greater potential to influence length of stay. According to some authors’ findings, these protocols use an existing time (period in which the patient waits to be seen by the physician) for an additional purpose^38^. Thus, at the time of the first medical appointment, the patient already has the test results, necessary for the diagnostic decision, reducing the period of time in which the patient remains in the ES. Since high permanence time is one of the factors that influence overcrowding, the potential of the ATP in reducing overcrowding in the ES stands out^45^. A relationship between long length of stay and increased patient mortality was also identified^9^ ^,^ ^46^.

Some studies also report that greater time reductions can be obtained when the mean length of stay of patients in the ES is high^40^ ^,^ ^47^. It was not possible to verify this data in this study because the mean ES time was only described in one study^36^.

Only two studies found statistical relevance in this outcome^36^ ^,^ ^38^. However, all the studies considered the time reductions found as clinically relevant, described as between 6.7 minutes^35^ and 56 minutes^36^.

Agreement between the diagnostic interventions initiated in the triage was reported in five studies, whether requested by nurses or by physicians. The relevance of this outcome was described in some studies, where the authors state that nurses are qualified to initiate diagnostic or therapeutic approaches in the triage^1^ ^,^ ^48^ ^-^ ^49^.

Even though they were not included in the search strategy of this SR, the outcomes related to the number of exams requested and the professionals’ and users’ satisfaction were analyzed, considering their representativeness and relevance for using advanced triage protocols in the ES. 

### Exam Requests

In this outcome, the comparative data between the number of exams requested and their positive findings were considered. This was the main result described by one of the authors, who stated that the triage orders do not compromise care safety in the ES^23^. This meta-analysis found no relevant difference in the number of tests requested between the intervention and control groups (OR 0.94[0.64.1.38]), described with moderate certainty of evidence, as it supports the hypothesis that triage professionals are competent to initiate diagnostic processes^12^. 

Some service administrators fear the economic impact that this intervention may cause, as there can be an increase in the number of exams requested^32^. Such assertion was contradicted by subsequent studies, which stated that the reduction in the length of stay of the patients in the ES and, consequently, in the costs of this length of stay with medications and care, would offset a possible increase in the exams requested^1^ ^,^ ^12^ ^,^ ^50^ ^-^ ^51^, a situation that, as described, was not verified in this study.

From a multicentric study, it was concluded that a longer training period of the ES triage professionals in using the ATP was associated with a lower number of exams requested^39^. Other studies described similar findings, associating better performance and safety of the triage professionals with better training programs^12^ ^,^ ^48^ ^,^ ^50^. Advanced practice Nursing stands out, where nurses who work in ES triage have technical competence and autonomy for decision-making^52^.

### Patients’ and professionals’ satisfaction

Analyzing the patients’ and the professionals’ satisfaction is a quality indicator for a health service^9^ ^,^ ^52^. Positive results related to this topic using the ATP were identified in a Canadian study by describing 94% satisfaction among the patients with the use of the ATP^52^ and in an SR conducted in 2017^12^. Of the four CRTs that presented such outcome in this systematic review, three pointed to increased satisfaction using the ATP, and one of them identified no difference between the groups. 

The satisfaction of the patients and professionals who seek the ES characterizes an important quality indicator, which is significantly impaired when the ES is overcrowded or has long permanence times^9^, in addition to the association of this data with a worse prognosis^46^. Thus, although brief, the reduced time periods contributed to the satisfaction of patients and professionals of the ES, findings that supported the clinical relevance of the result^35^ ^-^ ^36^ ^,^ ^39^.

### Quality of the studies

From the risk of bias analysis, the quality of the studies was generally considered as satisfactory in the studies included. The main risk of bias identified was related to masking. None of the studies included in this review masked the participants. This is due to the type of intervention, which makes blinding both of patients and of triage professionals unfeasible. This risk of bias has already been reported in other studies^12^; however, as they are protocols, this does not harm the study result. Also within the scope of the masking bias, some studies did not perform masking of the evaluators. This masking would improve the quality of the studies^21^. In addition to that, the way in which one of the studies presented the data led the authors to consider an attrition bias^33^. Two studies^37^ ^,^ ^42^ do not describe the concern with other possible biases and one study^39^ refers to certain bias in the training of the triage professionals that influenced the results found, considered a high risk for other biases. In one of the studies^42^, randomization was performed inappropriately, throughout the days of the week, a factor that justifies its exclusion; the other studies used computerized algorithms for randomization. 

As for the certainty of the evidence, the outcome of the length of stay of the patients in the ES presented very low certainty of evidence. The authors believe that this is due to the low number of CRTs included, to the use of different ATPs in each study, and to the lack of standardization in data presentation, requiring their conversion. This difficulty was reported in other SRs^12^ ^,^ ^45^. Due to greater clarity in data presentation, even if extracted from the same studies, moderate certainty of the evidence stands out for the outcome of exams requested.

Since only 10 CRTs were selected, the existence of some publication bias was considered, evidenced by the analysis of the funnel graph. The lack of standardization in the format of the data presented was also a limitation of this study, requiring conversion of medians to means in some studies, as well as the definition of confidence intervals, implying possible result bias.

## Conclusion

Using the advanced triage protocol in emergency services made it possible to attain a 36-minute mean reduction in the length of stay of patients in this locus, with greater repercussion in services with prolonged permanence times. The triage professionals are competent to initiate diagnostic procedures in triage in a safe manner, as long as they are trained to do so, and these measures reflect an increase in the patients’ satisfaction.

This study evidenced a guiding element for a change in behavior that contributes, through the optimization of care processes, to a cost-effective and safe reduction in the patients’ length of stay and overcrowding in the ES. 

However, the need to conduct good quality CRTs on the theme is highlighted, especially in ES with high permanence times, in order to obtain papers that are more methodologically adequate, as well as the need to develop more cost-effectiveness studies to determine the economic impact of using the advanced triage protocol
